# Dapagliflozin-associated euglycemic diabetic ketoacidosis in a patient with type 2 diabetes mellitus: A case report

**DOI:** 10.1097/MD.0000000000020228

**Published:** 2020-05-22

**Authors:** In Hee Lee, Dong Jik Ahn

**Affiliations:** a Department of Internal Medicine, Daegu Catholic University School of Medicine; b Department of Internal Medicine, Hansung Union Internal Medicine Clinic and Dialysis Center, Daegu, Republic of Korea.

**Keywords:** dapagliflozin, euglycemic diabetic ketoacidosis, sodium-glucose co-transporter-2 inhibitor

## Abstract

**Rationale:**

Rare cases of euglycemic diabetic ketoacidosis (eu-DKA) have been reported after the administration of sodium-glucose cotransporter-2 (SGLT-2) inhibitors. No reports have described eu-DKA complicated by hypernatremia due to SGLT-2 inhibitors.

**Patient concerns:**

A 76-year-old woman with a 40-year history of type 2 diabetes mellitus (DM), for which metformin (1000 mg/day) and dapagliflozin (10 mg/day) were prescribed, presented with malaise, fever, and oliguria. On presentation, her white blood cell count (11,800/μL), serum creatinine (3.2 mg/dL), and C-reactive protein (54 mg/L) were abnormal. Bilateral pyeloureteritis and diffuse paralytic ileus were present. She received intravenous antibiotics and total parenteral nutrition, and was asked to fast. Her renal function and ileus briefly improved. Oral hypoglycemic agents, metformin and dapagliflozin, along with enteral feeding were reinstituted on day 3 of hospitalization. However, on day 6 of hospitalization, the patient developed an altered state of consciousness including confusion, lethargy, and stupor. Several laboratory abnormalities suggestive of ketoacidosis with euglycemia were noted.

**Diagnoses:**

The patient was diagnosed with eu-DKA accompanied by severe hypernatremia (corrected serum Na^+^ concentration, 163 mEq/L) and hypokalemia following dapagliflozin re-administration.

**Interventions:**

The patient was treated with indicated intravenous fluid therapy. Dapagliflozin use was discontinued.

**Outcomes:**

The patient's mental status and laboratory findings improved gradually, and she was discharged on maintenance doses of insulin and metformin on day 14 of hospitalization.

**Lessons:**

Acute illnesses such as diffuse paralytic ileus and urinary tract infection, and dietary restrictions or fasting in patients with DM can be considered potential predisposing factors for SGLT-2 inhibitor-associated eu-DKA. For patients with diabetes in the setting of acute morbidity, timely resumption of the SGLT-2 inhibitor therapy should be carefully determined. In addition, eu-DKA due to SGLT-2 inhibitor use may be accompanied by electrolyte disturbances such as hypernatremia and hypokalemia.

## 1 Introduction

Sodium-glucose cotransporter-2 (SGLT-2) inhibitors are oral hypoglycemic agents that promote glycosuria by inhibiting renal SGLT-2 receptors responsible for glucose reabsorption.^[1]^ SGLT-2 inhibitors induce weight loss by promoting the urinary excretion of calories and inhibiting adipose tissue formation, thereby improving insulin sensitivity.^[1]^ Monotherapy with SGLT-2 inhibitors as insulin-independent hypoglycemic agents or combination therapy with other hypoglycemic agents for treating type 2 diabetes mellitus (DM) is increasing.^[1]^ Recently, however, there has been a gradual increase in reports on diabetic ketoacidosis (DKA) after administration of SGLT-2 inhibitors.^[2–6]^ Here, we report a 76-year-old female patient with type 2 DM who developed euglycemic diabetic ketoacidosis (eu-DKA) with hypernatremia following dapagliflozin re-administration.

## 2 Case report

**Figure 1:**
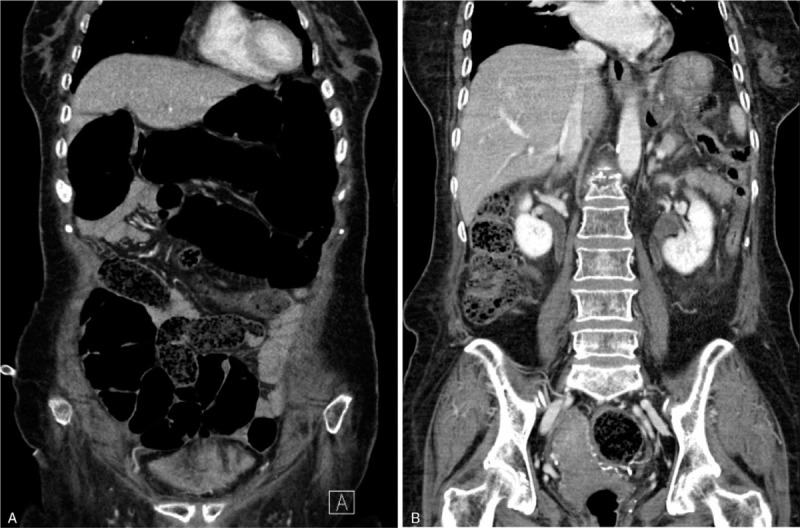
Abdominal computed tomography demonstrate paralytic ileus (A) and contrast enhancement along the renal pelvis and ureteral walls of both kidneys (B). Compression fracture of the first lumbar vertebra is also noted (B).

The patient was a 76-year-old woman who presented to our emergency room with a 3-day history of general weakness, fever, and oliguria and a 7-day history of nausea, and vomiting. The patient was bedridden due to a compression fracture of the 1st lumbar vertebra sustained 5 days prior to presentation. The patient had been taking appropriate oral medications for DM and hypertension for the past 40 years and had not developed any specific renal disease. Her prescribed oral hypoglycemic agents at the time of presentation were metformin (500 mg twice per day) and dapagliflozin (10 mg/day). Other daily medications included ezetimibe/rosuvastatin calcium (10 mg/5.2 mg), clopidogrel (75 mg), nicorandil (5 mg), imipramine (25 mg), tolterodine (4 mg), and tamsulosin (0.2 mg). Blood pressure, pulse rate, respiration rate, and body temperature at presentation were 160/80 mmHg, 120/min, 20/min, and 37.5°C, respectively. The patient was alert but experiencing malaise. Her oral mucosa was dry and skin turgor had decreased. Chest auscultation results were normal, but her abdomen was distended with decreased bowel sounds. A peripheral blood test at admission showed a white blood cell (WBC) count of 11,800/μL (neutrophils 85.3%), hemoglobin of 13 g/dL, a platelet count of 173,000/μL, and an erythrocyte sedimentation rate of 12 mm/hour. Serum biochemical examination showed the following: glucose, 410 mg/dL; blood urea nitrogen, 41.7 mg/dL; creatinine (Cr), 3.2 mg/dL; albumin, 3.8 g/dL; total cholesterol, 93 mg/dL; calcium, 8.1 mg/dL; phosphorus, 4.7 mg/dL; uric acid, 6.1 mg/dL; C-reactive protein, 54 mg/L; glycated hemoglobin (HbA_1C_), 8.1%; insulin, 3.3 μIU/mL (reference range, 2.6–24.9 μIU/mL); and C-peptide, 1 ng/mL (reference range, 1.1–4.4 ng/mL). Urinalysis with microscopic examination showed albumin 3+, occult blood 2+, a WBC count of 20–30/high power field, and a red blood cell count of 10–30/high power field (dysmorphic 90%). Spot urine protein to Cr ratio was 1.14 g/g. After urinary catheterization, her urine output was 1950 mL suggesting azotemia due to postrenal acute kidney injury. Abdominal computed tomography scan showed diffuse paralytic ileus and bilateral pyeloureteritis (Fig. 1A, 1B). Treatment with empirical broad-spectrum antibiotics (ceftriaxone) was initiated along with fluid therapy and total parenteral nutrition after insertion of a nasogastric tube. Fasting was prescribed. The oral hypoglycemic agents were discontinued. On day 3 of hospitalization, the patient was transitioned to a full-liquid diet through a nasogastric tube because she showed improvement in renal function (serum Cr, 0.7 mg/dL); paralytic ileus was also less severe. Treatment with metformin (1000 mg/day) and dapagliflozin (10 mg/day) was reinstituted. Previous outpatient medications were also retained and no new medications were added. Extended spectrum beta-lactamase negative, ceftriaxone–sensitive *Escherichia coli* was isolated from urine cultures. However, on day 6 of hospitalization, she developed consciousness alterations, including confusion, lethargy, and stupor, along with nausea, vomiting, and abdominal pain. Arterial blood gas analysis showed a pH of 6.904, partial pressure of carbon dioxide of 12.0 mmHg, and HCO_3_^−^ of 3.1 mmol/L, suggestive of high anion gap metabolic acidosis with respiratory compensation. Based on the results of serum biochemical examination (Table 1), we suspected eu-DKA accompanied by hypovolemia, hypernatremia, and hypokalemia. For the first 6 hours after discontinuation of dapagliflozin, we performed intravenous fluid therapy with 0.9% saline at a rate of 250 mL/hour (h) for 2 hours followed by 100 mL/h, 5% dextrose in water (5% D/W) (100 mL/h), and KCl (40 mEq/L). Regular insulin (RI) and sodium bicarbonate were not administered considering the blood glucose levels (range, 150–250 mg/dL) and arterial blood pH (>6.9). Because disturbances in serum electrolyte levels continued for 6 hours after intravenous fluid resuscitation (Table 1), administration of 0.45% saline (100 mL/h) with KCl (20 mEq/L), 5% D/W (50 mL/h), and RI (2.5 U/h, 0.05 U/kg/h) was maintained. Brain magnetic resonance imaging of the patient showed no specific abnormalities, including the absence of acute ischemic brain lesions. Eventually, the patient started enteral feeding of free water through a nasogastric tube while being administered a mixed solution of 0.45% saline and 5% D/W with RI. Additional biochemical results are described in Table 1. After the 8th day of hospitalization, consciousness and laboratory findings improved; however, dapagliflozin was not resumed. The patient was discharged on the 14th hospital day because of resolution of consciousness impairment and laboratory abnormalities, and the patient is currently receiving insulin (glargine/insulin lispro) and metformin for management of DM.

**Table 1:**
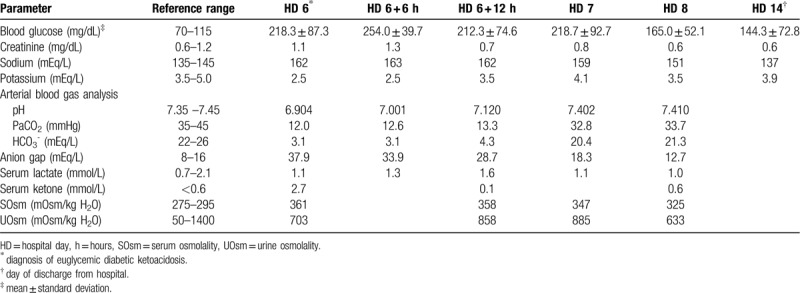
Laboratory data of the patient during hospitalization.

## 3 Discussion

SGLT-2 receptors are transporters that drive the reabsorption of approximately 90% of filtered glucose in the S1 segment of the proximal tubule in the kidney.^[7]^ Selective SGLT-2 inhibitors inhibit the SGLT-2 transporters, thereby preventing the reabsorption of glucose and reducing blood glucose levels by inducing glycosuria.^[7]^ In addition, these inhibitors are oral hypoglycemic agents with various clinical benefits, including improving insulin sensitivity by decreasing visceral and subcutaneous fat, decreasing the risk of cardiovascular mortality, and decreasing hypoglycemic episodes.^[7]^ Selective SGLT-2 inhibitors approved by the US Food and Drug Administration (FDA) and widely used for the treatment of type 2 DM include dapagliflozin, canagliflozin, and empagliflozin.^[7]^

DKA is a fatal acute complication of DM that occurs as the result of severe insulin deficiency. DKA is commonly associated with stress, such as that associated with infection or major surgery, in patients with type 2 DM.^[8]^ Eu-DKA is an uncommon form of DKA that is characterized by metabolic acidosis (pH < 7.3), a decreased level of serum bicarbonate (<18 mEq/L), and a relatively low blood glucose level (<200 mg/dL).^[9]^ The proposed mechanisms of eu-DKA induced by SGLT-2 inhibitors are as follows: SGLT-2 inhibitors reduce blood glucose levels, thereby decreasing the secretion of endogenous insulin by pancreatic β-cells. This in turn stimulates pancreatic α-cells, leading to increased glucagon secretion. SGLT-2 inhibitors also directly stimulate α-cells, thereby increasing plasma glucagon concentration and promoting hepatic ketogenesis.^[9]^ In addition, SGLT-2 inhibitors increase the reabsorption of acetoacetate in the renal tubules, which increases the blood level of ketone bodies.^[9]^

The incidence of DKA associated with dapagliflozin has been reported to be < 0.1%,^[10]^ but reports of DKA associated with SGLT-2 inhibitors have recently increased.^[2]^ To our best knowledge, there have been no published reports on the relationship between development of eu-DKA and dosage or duration of SGLT-2 inhibitor administration. A meta-analysis by Burke et al. included data on 34 patients who developed DKA due to SGLT-2 inhibitor use, of which 25 (73.5%) had type 2 DM. The average age of those presenting with DKA was 51.2 ± 16.3 years, and the average concentration of blood glucose was 265.6 ± 140.7 mg/dL (range, 68–500 mg/dL).^[3]^ The average values of pH and anion gap were 7.05 ± 0.15 and 21.6 ± 5.3 mEq/L, respectively. The predisposing factors for SGLT-2 inhibitor-associated DKA were latent autoimmune diabetes in adults, major surgeries, missed or discontinued insulin administration, inappropriate reduction of insulin dosage, acute medical illness, low-carbohydrate diet, acute pancreatitis, and dehydration.^[3]^ Common symptoms in these patients included abdominal pain, nausea, and vomiting; non-specific symptoms, such as tachycardia, general weakness, and impaired consciousness, were also noted.^[3]^ The patient in our case was a 76-year-old woman with a blood glucose level of 156 mg/dL, an arterial blood pH of 6.904, and a calculated anion gap of 37.9 mEq/L at presentation of eu-DKA (Table 1). The patient complained of abdominal pain, nausea, vomiting, and consciousness alterations; these manifestations were similar to those in patients reported previously.^[3]^ In this patient, following the resolution of acute azotemia and diffuse paralytic ileus, the administration of oral hypoglycemic agents including metformin and dapagliflozin along with enteral feeding was resumed. Except for sick-day protocol, there are no clear guidelines regarding the optimal timing for SGLT-2 inhibitors reinstitution in patients with acute illnesses that might precipitate ketoacidosis.^[11]^ Combination therapy with SGLT-2 inhibitors and metformin is not known as a risk factor for eu-DKA.^[3]^ However, diffuse paralytic ileus is often associated with anorexia, nausea and vomiting, which can decrease calorie or carbohydrate intake. Resultant calorie deficits can lower insulin secretion and enhance release of counter-regulatory hormones such as catecholamines, cortisol, glucagon, and growth hormone.^[12]^ Urinary tract infection in patients with type 2 DM can be accompanied by relative insulin deficiency, which leads to ketoacid formation through increase of lipolysis and free fatty acids.^[13]^ Concomitant elevation in serum counter-regulatory hormones promotes the urinary loss of glucose, resulting in euglycemia. An increase in plasma glucagon concentration also causes free fatty acid oxidation and ketone production.^[14]^ These possible mechanisms in combination with the use of SGLT-2 inhibitors can make diabetic patients more prone to develop eu-DKA.^[8,14]^ In our patient, urinary tract infection, dietary restriction, and fasting for therapeutic purposes, as well as non-utilization of insulin were thought to be the predisposing factors for the development of eu-DKA. Early resumption of dapagliflozin administration following these relevant clinical settings may have resulted in apparent eu-DKA.

If eu-DKA is suspected, it is necessary to rule out other conditions inducing high anion gap metabolic acidosis, such as lactic acidosis, toxic alcohol (methanol or ethylene glycol) intoxication, drug abuse, and oliguric renal failure.^[15]^ Our patient could have easily been diagnosed with starvation ketoacidosis, but this presumptive diagnosis could be excluded based on the laboratory findings, including the considerably low arterial blood pH (6.904) and low level of serum bicarbonate (3.1 mEq/L).^[15]^ Treatment of SGLT-2 inhibitor-associated eu-DKA is similar to that of conventional DKA, apart from the prompt discontinuation of SGLT-2 inhibitors: parenteral infusion of resuscitation fluids, insulin administration, and correction of electrolytic disturbances are recommended until the serum anion gap has normalized.^[15]^ Caution should be exercised with re-administration of SGLT-2 inhibitors after eu-DKA has improved.

Cases of hypernatremia in adult patients with DKA or patients with type 2 DM receiving SGLT-2 inhibitors have been reported previously.^[16–18]^ However, no reports have described eu-DKA complicated with hypernatremia due to SGLT-2 inhibitors use as in this case. SGLT-2 inhibitors reduce the reabsorption of sodium in the renal tubule, leading to mild temporary diuresis.^[18]^ Increased glucose load in the renal tubule due to constant glycosuria causes osmotic diuresis, thereby promoting the excretion of hypotonic urine and eventually leading to dehydration and hypovolemic hypernatremia. In addition, decreased circulating volume in the intravascular space induces hyperaldosteronism, leading to further exacerbation of hypernatremia and hypokalemia.^[19,20]^ When the SGLT-2 receptor is blocked, flow rate increases in the distal tubule, leading to urinary potassium loss as well.^[20]^ In our case, analysis of urine electrolytes showed a urine Na^+^/K^+^ concentration of 16/34.1 mEq/L and a urine Cr of 64.5 mg/dL at presentation along with a spot urine K^+^ to Cr ratio of 52.9 mEq/g. Fractional excretion of sodium and fractional excretion of potassium were calculated to be 0.2% and 27.5%, respectively. These findings suggest effective hypovolemia due to sustained osmotic diuresis and urinary potassium loss as the result of secondary hyperaldosteronism.^[21,22]^ Accordingly, we postulated that eu-DKA, severe hypernatremia (corrected serum Na^+^ concentration, 163 mEq/L), and hypokalemia in this patient were substantially associated with administration of dapagliflozin.^[20]^ The Naranjo adverse drug reaction assessment score applied to this case was 6 points, suggesting a probable drug reaction to dapagliflozin.^[23]^ Acute hypernatremia can induce severe cellular dehydration in the central nervous system (CNS), leading to altered mental status and lethargy. The first-line treatment of these manifestations is correction of the underlying disease and administration of hypotonic fluid.^[24]^ Relatively slow correction of hypernatremia (<8–10 mEq/L per day) is recommended because a rapid decrease in serum sodium concentration can cause cerebral edema that leads to fatal CNS complications such as seizures, cerebral herniations, and death.^[24]^ Our patient was administered a solution of 0.45% saline with KCl and 5% D/W with RI parenterally in addition to enteral feeding of free water through a nasogastric tube over 3 to 4 days. Her CNS symptoms and malaise improved gradually as hypovolemic hypernatremia and hypokalemia were steadily corrected.

In summary, we report a case of a 76-year-old woman with type 2 diabetes who developed eu-DKA following dapagliflozin re-administration. The findings of this case suggest that acute illnesses such as diffuse paralytic ileus and urinary tract infection, and dietary restrictions or fasting in patients with DM can be potential predisposing factors for the development of SGLT-2 inhibitor-associated eu-DKA. Therefore, in diabetic patients with these clinical conditions, timely resumption of SGLT-2 inhibitor therapy should be considered. In addition, eu-DKA due to SGLT-2 inhibitor use may be accompanied by electrolyte disturbances, such as hypovolemic hypernatremia and hypokalemia, necessitating an appropriate fluid therapy and close monitoring.

## Author contributions

**Conceptualization:** In Hee Lee.

**Data curation:** In Hee Lee, Dong Jik Ahn.

**Formal analysis:** In Hee Lee.

**Methodology:** In Hee Lee.

**Validation:** Dong Jik Ahn.

**Writing – original draft:** In Hee Lee.

**Writing – review & editing:** In Hee Lee.

In Hee Lee orcid: 0000-0003-3562-7586.
